# Trends in the Comprehension and Management of Gastrointestinal Tract Disorders

**DOI:** 10.3390/jcm11061730

**Published:** 2022-03-21

**Authors:** Marilena Durazzo, Arianna Ferro, Sharmila Fagoonee, Rinaldo Pellicano

**Affiliations:** 1 Department of Medical Sciences, University of Turin, C.so A.M. Dogliotti 14, 10126 Turin, Italy; direzione.scienzemediche@unito.it; 2 Institute for Biostructure and Bioimaging, National Research Council, Molecular Biotechnology Centre, 10126 Turin, Italy; sharmila.fagoonee@unito.it; 3 Unit of Gastroenterology, Città della Salute e della Scienza Hospital, C.so Bramante 88, 10126 Turin, Italy; rinaldo_pellican@hotmail.com

During the last decade, relevant advances have been made in the knowledge of the pathogenetic mechanisms of gastrointestinal (GI) tract disorders. This has led to a better management of these morbidities that, regarding the healthcare required for a longer lifespan, represent a significant burden for all national health systems around the world [1]. In fact, aging increases the risk for chronic and neoplastic diseases as well as the worsening of already existent disturbances [2].

The epidemiological changes that have occurred lately have been influenced by the improved management of some health conditions, as well as exogenous factors. For instance, after the introduction of new drugs and new strategies for the cure of *Helicobacter pylori* infection—particularly, the single-capsule bismuth quadruple therapy [3] and the use of tailored approaches [4]—a drastic reduction in terms of the incidence and prevalence of this infection as well as other related morbidities (peptic ulcer disease) is expected [5]. On the other hand, the increasing prevalence of a more “Westernized” lifestyle (including dietary changes and a decrease in physical activity) has been associated with a diet rich in fat and protein, and a rise in the incidence of gastroesophageal reflux disease (GERD), which affects esophageal and extra-esophageal systems [6]. In parallel, this change in lifestyle has been associated with an increased incidence of metabolic-associated fatty liver disease (MAFLD) [7], which in the clinical setting, reflects in a shift from a higher prevalence of viral liver diseases to a higher prevalence of dysmetabolic liver diseases [8], as well as a significant increase in the incidence of malignancies as colorectal cancer (CRC). Within this negative context, the increased incidence of disorders caused by inappropriate alcohol consumption [9], associated with both hepatic and extra-hepatic alcohol-related disorders play a major role [10]. Since modifiable factors can be corrected after the appropriate education and psychological support, carrying out this task has become a priority.

Inflammatory bowel diseases (IBD) represent another prime example that highlights therapeutic improvements of GI tract disorders. The introduction, in the clinical setting, of biologic drugs has allowed the management of IBD patients to become optimized for steroid-refractory or steroid-resistant diseases [11], improving not only the mucosal state (with a mucosal remission) but also the clinical consequences of this inflammation [12]. These advances have allow us to focus on the endpoint beyond the clinical and endoscopic parameters, including the patient’s quality of life [13].

Increasingly often, the interdisciplinary aspect of GI pathologies is the object of studies that aim to optimize the management of patients with complex diseases, a prime example being the management of GI conditions in diabetic patients. Nevertheless, despite the intense efforts made from basic research [14] to the clinical setting [15], diabetic gastroparesis remains a challenge for clinicians. The involvement of the hepato-pancreato-biliary tract in the context of autoimmune manifestations is another key example of this [16]. While some diseases, such as autoimmune hepatitis and primary biliary cholangitis, are well-known, others, such as autoimmune pancreatitis, represent a challenge for clinicians in several fields (gastroenterologists, experts in endoscopic or radiologic imaging, and immunologists). In the presence of these conditions, a multidisciplinary approach is essential to determine both the appropriate diagnosis and optimal treatment for managing patients, whilst avoiding undervaluation, overmedicalization and unnecessary costs.

The emergence of microbiota–microbiome investigations into the spotlight has opened a door to several research possibilities and could, in theory, help to offer therapeutic interventions for a broad series of GI diseases. These interventions could range from benign, non-inflammatory [17] or inflammatory types [18] to malignant diseases [19].

Finally, the current pandemic, caused by severe acute respiratory syndrome coronavirus-2 (SARS-CoV-2), has drastically impacted human society, causing not only diseases of the respiratory tract [20] but also of the digestive tract [21]. Furthermore, some GI conditions, such as bleeding and the increased the risk of death among patients with coronavirus disease-19 (COVID-19), were discovered [22]. Patients affected by COVID-19 also experienced an increase in GI symptoms, but this was not associated with hospitalization or mortality rates [23]. This could be due to the fact that a relevant part of these manifestations were likely to have had an anxiety-induced functional basis. This has also been associated with GI disturbances reported by medical students during the COVID-19 lockdown periods [24].

## Data Availability

Not applicable.

## References

[1] Peery A.F., Crockett S.D., Murphy C.C., Jensen E.T., Kim H.P., Egberg M.D., Lund J.L., Moon A.M., Pate V., Barnes E.L. (2022). Burden and Cost of Gastrointestinal, Liver, and Pancreatic Diseases in the United States: Update 2021. Gastroenterology.

[2] Durazzo M., Campion D., Fagoonee S., Pellicano R. (2017). Gastrointestinal tract disorders in the elderly. Minerva Med..

[3] Nyssen O.P., Perez-Aisa A., Castro-Fernandez M., Pellicano R., Huguet J.M., Rodrigo L., Ortuñ J., Gomez-Rodriguez B.J., Pinto R.M., Areia M. (2021). European Registry on Helicobacter pylori management: Single-capsule bismuth quadruple therapy is effective in real-world clinical practice. United Eur. Gastroenterol. J..

[4] Choe A.R., Shim K.N., Park Y., Song E.M., Tae C.H., Jung S. (2021). Cost-Effectiveness, Efficacy, and Safety Analysis of Tailored Therapy in Patients with Helicobacter pylori Infection. J. Clin. Med..

[5] Mladenova I. (2021). Clinical Relevance of *Helicobacter pylori* Infection. J. Clin. Med..

[6] Durazzo M., Lupi G., Cicerchia F., Ferro A., Barutta F., Beccuti G., Gruden G., Pellicano R. (2020). Extra-Esophageal Presentation of Gastroesophageal Reflux Disease: 2020 Update. J. Clin. Med..

[7] Eslam M., Sanyal A.J., George J., on behalf of the International Consensus Panel (2020). MAFLD: A Consensus-Driven Proposed Nomenclature for Metabolic Associated Fatty Liver Disease. Gastroenterology.

[8] Saracco G.M., Evangelista A., Fagoonee S., Ciccone G., Bugianesi E., Caviglia G.P., Abate M.L., Rizzetto M., Pellicano R., Smedile A. (2016). Etiology of chronic liver diseases in the Northwest of Italy, 1998 through 2014. World J. Gastroenterol..

[9] Testino G., Fagoonee S., Caputo F., Pellicano R. (2021). The early identification of alcohol use disorders and liver injury: Proposal for a diagnostic algorithm. Panminerva Med..

[10] Rosa G.M., Scagliola R., Zoppoli G., Perna V., Buscaglia A., Berri A., DELLA Bona R., Porto I., Pellicano R., Testino G. (2022). May standard basal echocardiogram allow to obtain predictors of asymptomatic cardiac dysfunction in alcoholics?. Minerva Med..

[11] Bertani L., Caviglia G.P., Antonioli L., Pellicano R., Fagoonee S., Astegiano M., Saracco G.M., Bugianesi E., Blandizzi C., Costa F. (2020). Serum Interleukin-6 and -8 as Predictors of Response to Vedolizumab in Inflammatory Bowel Diseases. J. Clin. Med..

[12] Scarozza P., De Cristofaro E., Scucchi L., Rocchetti I., Marafini I., Neri B., Salvatori S., Biancone L., Calabrese E., Monteleone G. (2020). Effect of Vedolizumab on Anemia of Chronic Disease in Patients with Inflammatory Bowel Diseases. J. Clin. Med..

[13] Sochal M., Małecka-Panas E., Gabryelska A., Talar-Wojnarowska R., Szmyd B., Krzywdzińska M., Białasiewicz P. (2020). De-terminants of sleep quality in inflammatory bowel diseases. J. Clin. Med..

[14] Ha C., Kim H., Cha R., Lee J., Lee S., Ryu J.H., Lee O.J. (2021). Effect of DA-9701 on the Gastrointestinal Motility in the Strepto-zotocin-Induced Diabetic Mice. J. Clin. Med..

[15] Bonetto S., Gruden G., Beccuti G., Ferro A., Saracco G., Pellicano R. (2021). Management of Dyspepsia and Gastroparesis in Patients with Diabetes. A Clinical Point of View in the Year 2021. J. Clin. Med..

[16] Kunovsky L., Dite P., Jabandziev P., Kala Z., Vaculova J., Andrasina T., Hrunka M., Bojkova M., Trna J. (2021). Autoimmune Diseases of Digestive Organs—A Multidisciplinary Challenge: A Focus on Hepatopancreatobiliary Manifestation. J. Clin. Med..

[17] Caviglia G.P., Tucci A., Pellicano R., Fagoonee S., Rosso C., Abate M.L., Olivero A., Armandi A., Vanni E., Saracco G.M. (2020). Clinical Response and Changes of Cytokines and Zonulin Levels in Patients with Diarrhoea-Predominant Irritable Bowel Syndrome Treated with *Bifidobacterium Longum* ES1 for 8 or 12 Weeks: A Preliminary Report. J. Clin. Med..

[18] Larussa T., Abenavoli L., Fabiano G., Mancuso M.A., Polimeni N., Dumitrascu D.L., Luzza F. (2021). Gut microbiota in in-flammatory bowel disease: A target for therapy not to be missed. Minerva Gastroenterol..

[19] Nouri R., Hasani A., Asgharzadeh M., Sefidan F.Y., Hemmati F., Rezaee M.A. (2022). Roles of gut microbiota in colorectal car-cinogenesis providing a perspective for early diagnosis and treatment. Curr. Pharm. Biotechnol..

[20] Koenigkam-Santos M., Wada D.T., Benatti M.N., Siyuan L., Batah S.S., Cetlin A.A., de Menezes M.B., Fabro A.T. (2022). SARS-Cov-2 pneumonia phe-notyping on imaging exams of patients submitted to minimally invasive autopsy. Ann. Transl. Med..

[21] Burkett A.E., Sher S.B., Patel C.R., Ildin-Eltoum I., Dhall D., Margaroli C., Peter S., Lee G., Bajpai P., Benson P.V. (2022). Gastrointestinal Manifestations of COVID-19 Infection: Clinicopathologic Findings in Intestinal Resections Performed at Single Institution. Front. Med..

[22] Zuin M., Rigatelli G., Fogato L., Zuliani G., Roncon L. (2021). Higher risk of death in COVID-19 patients complicated by gastroin-testinal bleeding events: A meta-analysis. Minerva Gastroenterol..

[23] Wang Y., Li Y., Zhang Y., Liu Y., Liu Y. (2022). Are gastrointestinal symptoms associated with higher risk of Mortality in COVID-19 patients? A systematic review and meta-analysis. BMC Gastroenterol..

[24] Abenavoli L., Cinaglia P., Lombardo G., Boffoli E., Scida M., Procopio A., Larussa T., Boccuto L., Zanza C., Longhitano Y. (2021). Anxiety and Gastrointestinal Symptoms Related to COVID-19 during Italian Lockdown. J. Clin. Med..

